# Supplementary material for: A coregulatory influence map of glioblastoma heterogeneity and plasticity
Source: NPJ Precis Oncol. 2025 Apr 15;9:110. doi: 10.1038/s41698-025-00890-0 (PMC12000621; doi:10.1038/s41698-025-00890-0)
Supplement: Supplementary file 9 — Supplementary information [file 41698_2025_890_MOESM9_ESM.pdf]

Supplementary Information for

A coregulatory influence map of glioblastoma heterogeneity and plasticity

This file includes:

- Supplementary Tables 1 to 4
- Supplementary Figures 1 to 4
- Legends for Supplementary Data 1 to 8

	Dataset	Publication ref.	Accession number	Platform	Samples
Metacohort Patient Tumor data	**Bao**	Genome Res. 2014	GSE48865	Illumina HiSeq 2000	100
	**Donson**	J Immunol. 2012	GSE33331	Affymetrix HG-U133 Plus 2.0	21
	**Ducray**	Mol Cancer. 2010	E-TABM-898	Affymetrix HG-U133 Plus 2.0	48
	**Gill**	PNAS 2014	GSE59612	Illumina HiSeq 2000	75
	**Gravendeel**	Cancer Res. 2009	GSE16011	Affymetrix HG-U133 Plus 2.0	159
	**Grzmil**	Cancer Res. 2011	GSE15824	Affymetrix HG-U133 Plus 2.0	15
	**Ivy_GAP**	Science. 2018	GSE107560	Affymetrix GWH SNP 6.0	54
	**Kwom**	PLoS One. 2015	GSE62153	Illumina HumanHT-12 V4.0	43
	**Li**	Oncotarget. 2014	GSE60184	Affymetrix HG-U133 Plus 2.0	23
	**Murat**	J Clin Oncol. 2008	GSE7696	Affymetrix HG-U133 Plus 2.0	80
	**Oh**	PLoS One. 2014	GSE58399	Affymetrix Human Gene 1.0 ST	105
	**Reifenberger**	Int J Cancer. 2014	GSE53733	Affymetrix HG-U133 Plus 2.0	70
	**Rembrandt**	Mol Cancer Res. 2009	GSE108476	Affymetrix HG-U133 Plus 2.0	219
	**TCGA_GBM**	Nature. 2008	Firehose 2016_01_28	HG-UG133A / Agilent-4502A	156/366
	**Vital**	Neuro Oncol. 2010	GSE43289	Affymetrix HG-U133 Plus 2.0	26
	**Walsh**	Mol Cancer Res. 2015	GSE51062	Affymetrix HG-U133 Plus 2.0	52
Cell lines	**CCLE**	Nature. 2019	19Q4 DepMap data	Illumina HiSeq 2000	42

Supplementary Table 1: Transcriptomic datasets used to generate GBM-cRegMap reference compounds. Related to Fig. 1.

	PamR (expression)		PamR (Influence)	
**Dataset**	**MCC**	**AUC**	**MCC**	**AUC**
**Bao**	-0,00000239	0,63	0,00000086	0,89
**Donson**	0,00000014	0,69	0,00000070	0,87
**Ducray**	-0,00000421	0,72	0,00000092	0,91
**Gill**	-0,00000206	0,62	0,00000079	0,86
**Gravendeel**	-0,00005709	0,67	0,00000095	0,92
**Grzmil**	-0,00000306	0,61	-0,00000040	0,82
**Ivy_Gap**	-0,00000373	0,65	0,00000085	0,89
**Kwom**	-0,00000143	0,71	0,00000087	0,89
**Li**	-0,00000089	0,70	-0,00000092	0,85
**Oh**	-0,00000825	0,70	0,00000093	0,91
**Reifenberger**	-0,00000413	0,72	0,00000094	0,92
**Rembrandt**	-0,00000104	0,70	0,00000088	0,89
**TCGA_Agilent**	-0,00000283	0,62	0,00000096	0,94
**TCGA_RSeq**	-0,00000180	0,71	0,00000095	0,93
**Vital**	-0,00004278	0,68	0,00000090	0,89
**Walsh**	-0,00000332	0,67	0,00000089	0,89

Supplementary Table 2: : Cross-study prediction performance for the resulted classifier of Verhaak subtypes using gene expression or influence in cross-batch prediction. Related to Fig. 2D. MCC: Matthews Correlation Coefficient. AUC: Area Under Curve. Used classifier: PAMR (prediction analysis for microarrays).

		PN	PN-L	NL	CL-A	CL-B	CL-C	MES
**Gender**	Female	75	27	26	71	37	63	79
	Male	104	40	51	88	63	94	189
	Ratio(F/M)	0,72	0,68	0,51	0,81	0,59	0,67	0,42
**Age**	<60	147	51	61	94	62	83	165
	[60,80]	53	16	37	75	57	80	109
	80+	4	2	1	2	2	6	8

Supplementary Table 3: Gender and age differences in GBM-cRegMap subclasses. Related to Fig. 3G.

Sample	Verhaak	cRegMap	PN	PN-L	NL	CL-A	CL-B	CL-C	MES
U87MG_1	MES	MES	0,06	0,01	0,02	0,06	0,10	0,05	0,69
U87MG_2	MES	MES	0,02	0,01	0,01	0,02	0,04	0,02	0,89
U87MG_3	MES	MES	0,02	0,01	0,01	0,02	0,04	0,02	0,89
U87MGR50_1	PN	CL-C	0,01	0,13	0,02	0,01	0,21	0,61	0,01
U87MGR50_2	CL	CL-C	0,01	0,05	0,01	0,02	0,05	0,86	0,00
U87MGR50_3	PN	CL-C	0,00	0,15	0,03	0,01	0,14	0,67	0,01
U87MGOFF_1	PN	PN	0,88	0,01	0,01	0,03	0,05	0,01	0,01
U87MGOFF_2	CL	PN	0,97	0,00	0,00	0,01	0,01	0,01	0,00
U87MGOFF_3	CL	PN	0,89	0,01	0,01	0,04	0,03	0,01	0,01

Supplementary Table 4: Classification of studied U87MG TMZ-resistant variants inhouse dataset (GSE253458). Related to Fig. 6B.

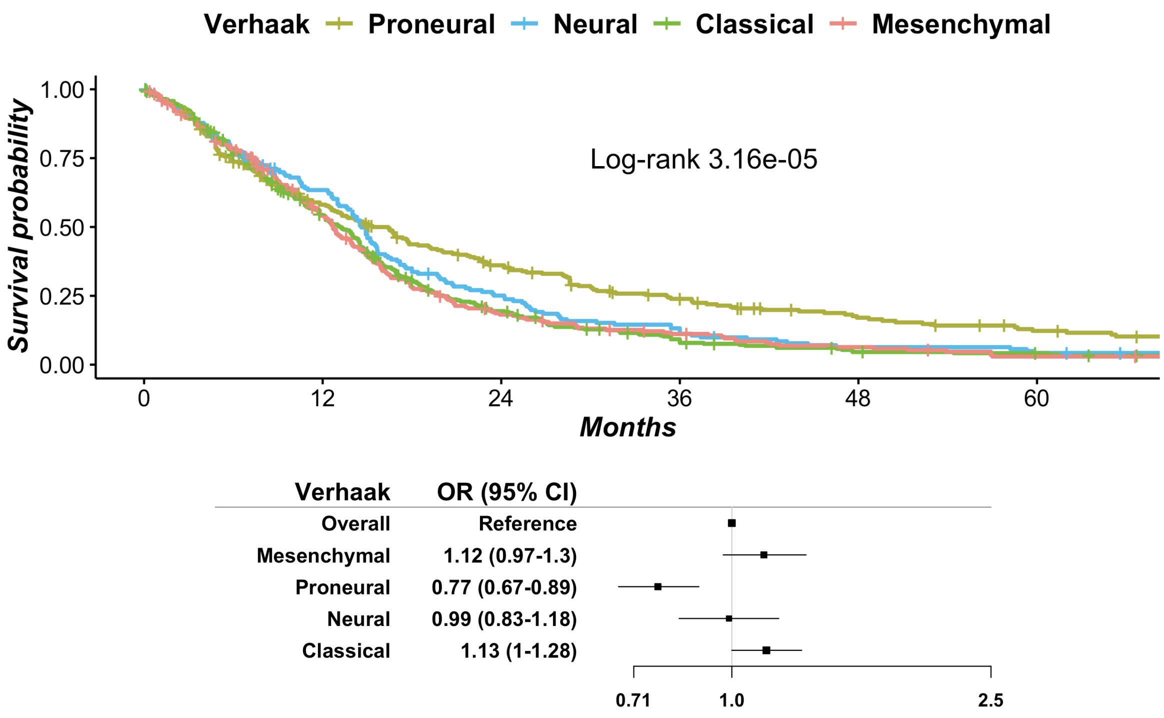

Supplementary Figure 1: Survival curve and forest plot of Verhaak classification. Survival analysis was performed with Cox regression model and displayed with survival R package. Overall metacohort's survival rate is taken as reference to allow cross-classification comparisons. Results are presented as hazard ratio with 95% confidence interval. Survival analysis was performed with Cox regression model with survival R package.

A

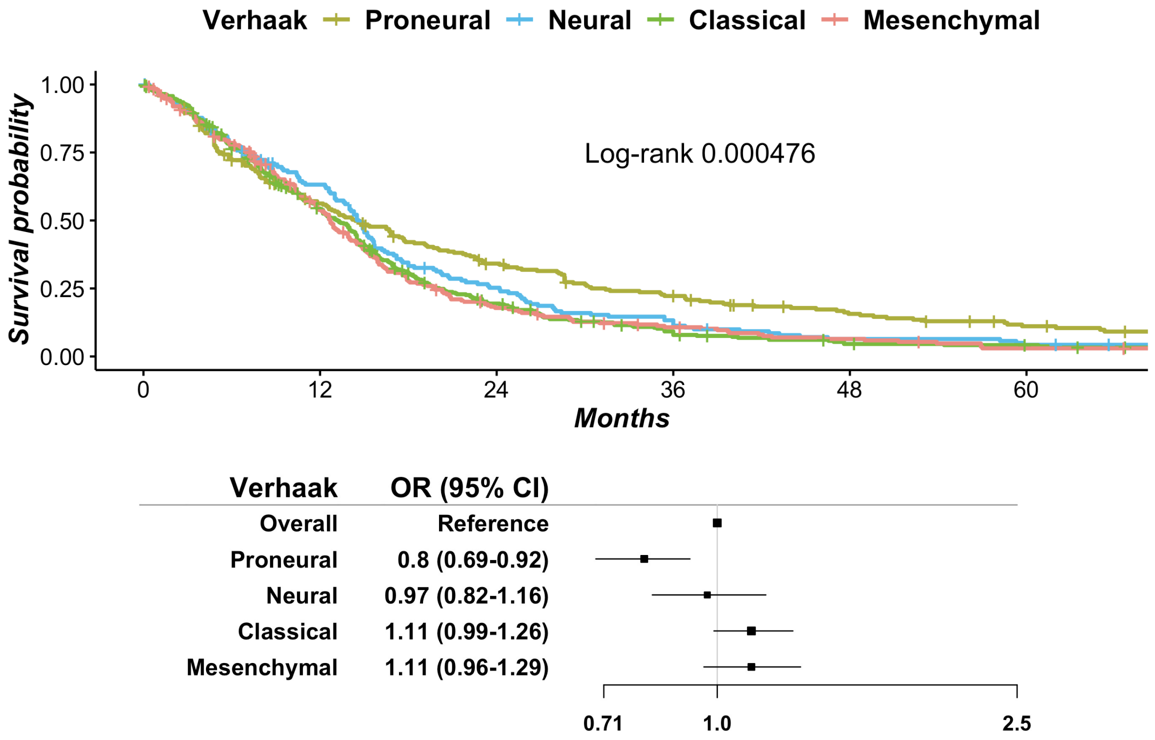

B

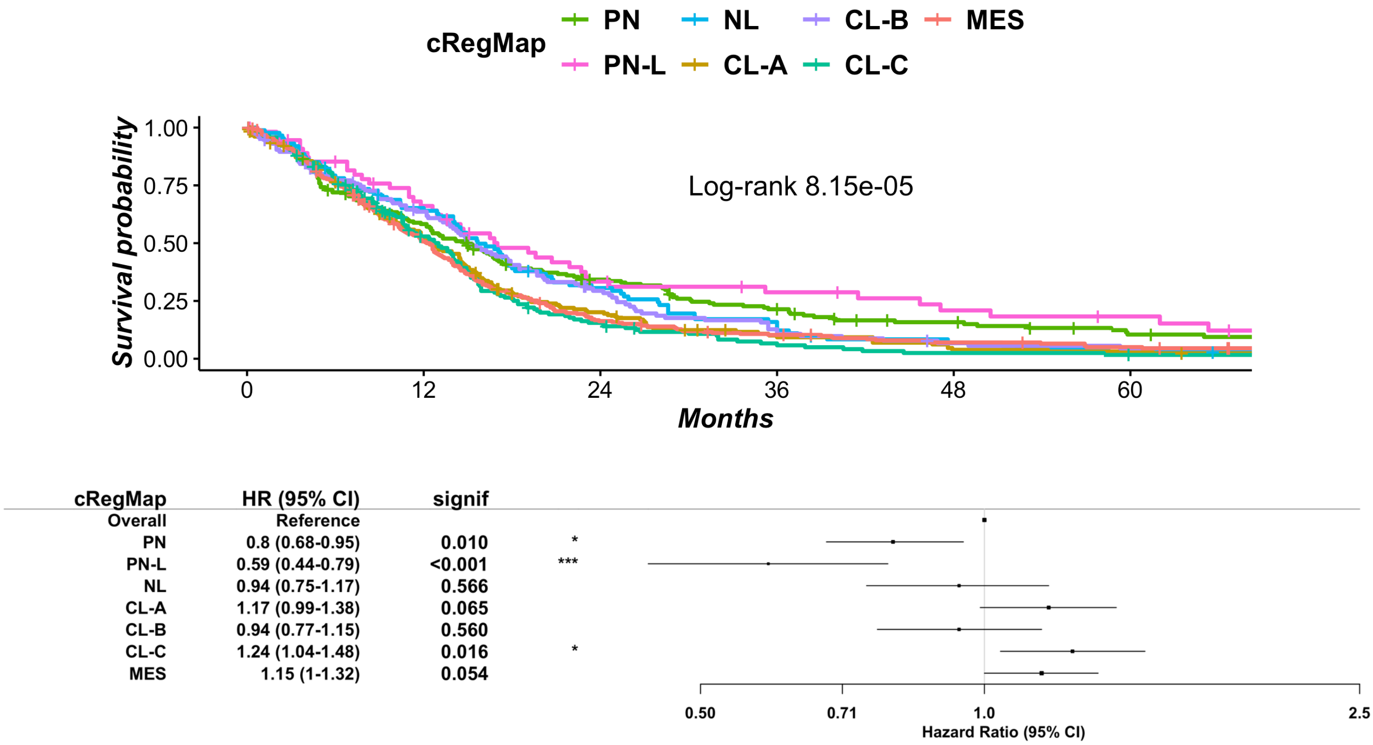

Supplementary Figure 2: Overall survival curve and forest plot of cRegMap and Verhakk classifications for non-IDH1-mutated samples. Survival curves and forest plot of cRegMap classification (A) and Verhaak classification (B) without IDH1 mutated samples. Survival analysis was performed with Cox regression model and displayed with survival R package. Overall metacohort's survival rate is taken as reference to allow cross-classification comparisons. Results are presented as hazard ratio with 95% confidence interval.

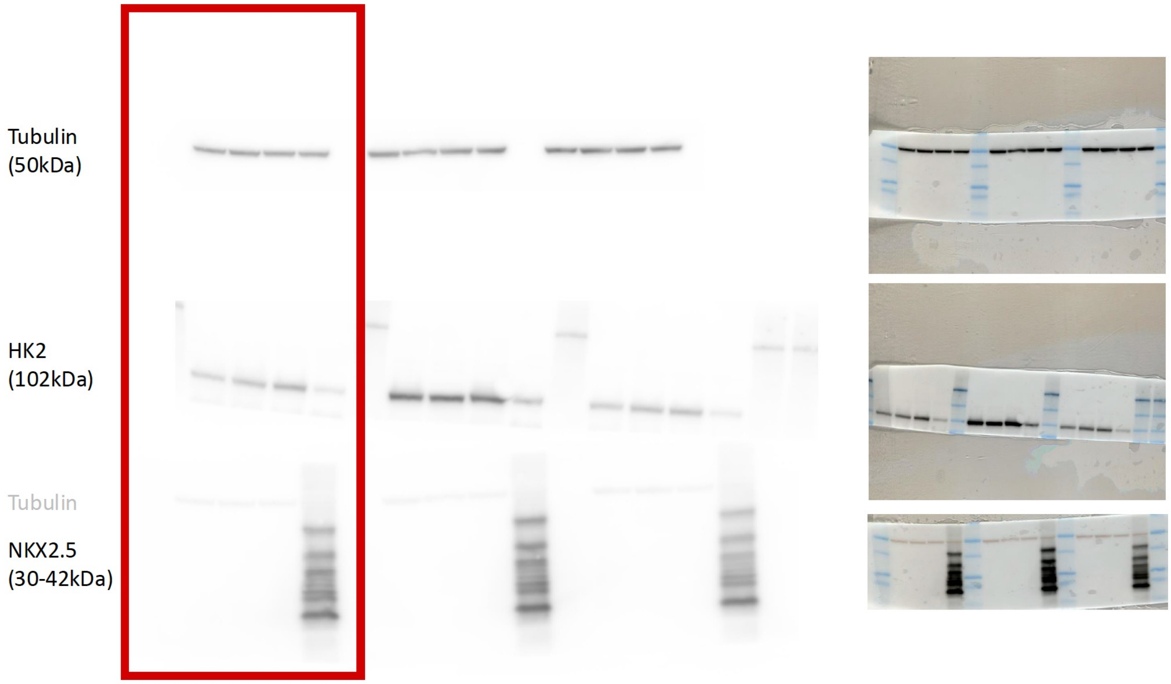

Supplementary Figure 3: Western blot analysis of PAX8 and GAPDH expression in U87MG, U87MG-R50, and U87MG-OFF cell lines. Primary antibodies were used against PAX8 (48–58 kDa) and GAPDH (36 kDa), with GAPDH serving as a loading control. Unprocessed blots are shown. Each blot is presented in two parts: the first displays the target protein, while the second corresponds to the molecular weight marker (ladder). Related to Fig. 7A in the main manuscript.

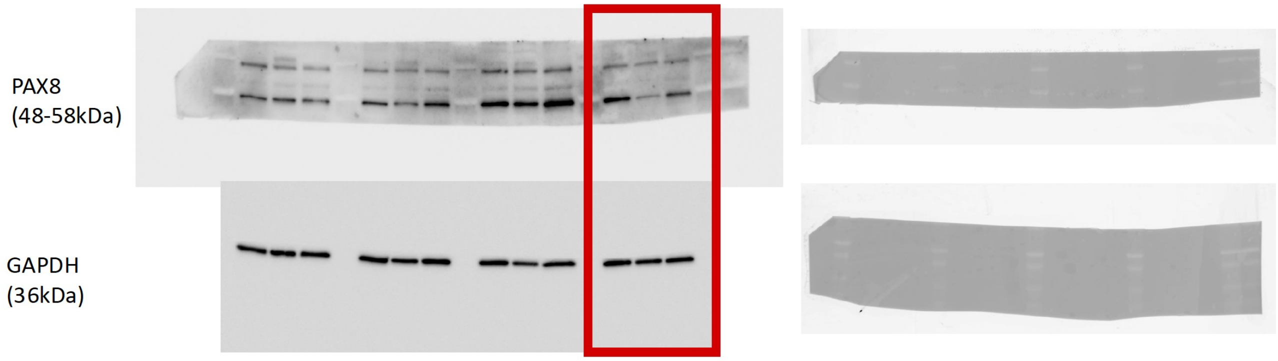

Supplementary Figure 4: Western blot analysis of NKX2-5, HK2, and Tubulin expression in U87MG control, Lipofectamine-treated, negative plasmid, and NKX2-5 plasmid-transfected cells. Primary antibodies were used against NKX2-5 (30–42 kDa), HK2 (102 kDa), and Tubulin (50 kDa), with Tubulin serving as a loading control. Unprocessed blots are shown. Each blot is presented in two parts: the first displays the target protein, while the second corresponds to the molecular weight marker (ladder). Related to Fig. 7E in the main manuscript.

Supplementary Data

Supplementary Data 1: Software, tools and resources required to develop GBM-cRegMap. Related to Fig. 1

Supplementary Data 2: GBM-CoRegNet regulators. Related to Fig.2A.

Supplementary Data 3: GBM-coRegNet co-regulators pairs. Related to Fig.2A.

Supplementary Data 4: Differentially expressed genes (DEGs) analysis using TCGA expression data with the Limma R package (adj. P <0.05 and logFC > 0.5). Related to Fig.3C.

Supplementary Data 5: Functional enrichment analysis of the DEGs (Supplementary Data 4) subclasses using clusterProfiler (GO-BP). Top 100 terms shown for each class. Related to Fig. 3C.

Supplementary Data 6: Differentially influenced regulators (DIRs) analysis. Related to Fig. 4A. DIRs extracted using Seurat::findallmarkers function (cutoff: min.pct: 0.25 and logfc.threshold: 0.20, adj. p-value <0.05). Additional ranking of DIRs (p-value (CERES)) was determined using the Wilcoxon-Mann-Whitney test between the average CERES dependency score of the cell lines assigned to the subclass (Supplementary Data 7) versus the average dependency score of the rest. The CERES dependency score of the cell lines was acquired using R package depmap.

Supplementary Data 7: Classification of glioblastoma cell lines. Related to Fig. 4B.

Supplementary Data 8: Functional enrichment analysis of the repressed and activated genes of NKX2.5 TF using clusterProfiler (GO-BP). Related to Fig. 7D
